# Innovating Technology-Enhanced Interventions for Youth Suicide: Insights for Measuring Implementation Outcomes

**DOI:** 10.3389/fpsyg.2021.657303

**Published:** 2021-06-03

**Authors:** Hannah S. Szlyk, Jia Tan, Rebecca Lengnick-Hall

**Affiliations:** ^1^School of Social Work, Rutgers, The State University of New Jersey, New Brunswick, NJ, United States; ^2^Brown School of Social Work, Washington University in St. Louis, St. Louis, MO, United States

**Keywords:** youth, suicidality, technology, psychosocial intervention, implementation science

## Abstract

Technology is one medium to increase youth engagement, especially among underserved and minority groups, in suicide preventive interventions. Technology can be used to supplement or adjunct an in-person intervention, guide an in-person intervention, or be the stand-alone (automated) component of the intervention. This range in technological use is now called the continuum of behavioral intervention technologies (BITs). Overall, suicide intervention researchers do not use this terminology to categorize how the role of technology differs across technology-enhanced youth interventions. There is growing recognition that technology-enhanced interventions will not create substantial public health impact without an understanding of the individual (youth, families, and providers), mezzo (clinics and health systems of care), and contextual factors (society, culture, community) that are associated with their implementation. Implementation science is the study of methods to promote uptake of evidence-based practices and policies into the broader health care system. In this review, we incorporate work from implementation science and BIT implementation to illustrate how the study of technology-enhanced interventions for youth suicide can be advanced by specifying the role of technology and measuring implementation outcomes.

## Introduction

Globally, suicide is a leading cause of death among youth (Centers for Disease Control and Prevention, National Center for Injury, Prevention and Control, 2020; World Health Organization, 2019). Suicidality, which includes suicidal ideation, plans of a suicide attempt, and actual suicide attempts (Posner et al., 2007) is also a pervasive problem that burdens young lives (Kokkevi et al., 2012; Page et al., 2013; Kann et al., 2018). Yet, many youth exhibiting suicidal thoughts and behaviors do not have contact with a mental health specialist over the course of a year, especially youth who identify as male, or as a racial or ethnic minority (Husky et al., 2012). Attitudinal (e.g., concerns about stigma, preference for self-management) and structural (e.g., limited time, transportation, and insurance) barriers may impact youth initiation of mental health services (Arria et al., 2011; Czyz et al., 2013). Untreated suicidality may lead to future psychological distress, as the presence of suicidal ideation during adolescence increases the odds of a suicide attempt in adulthood (Cha et al., 2017). Therefore, it is crucial that at risk individuals and those presenting symptoms of suicidality are engaged in appropriate mental health services as soon as possible (Cho et al., 2013).

As internet and smart-phone use is prominent among youth world-wide (Anderson and Jiang, 2018; Taylor and Silver, 2019), interventions that integrate technology may address barriers to engagement in in-person mental health services, such as access, reach, and stigma (Kreuze et al., 2017; Berrouiguet et al., 2018). Within suicidology, there has been a recent focus on the development of evidence-based technology-enhanced interventions and tools that integrate various technologies ranging from telephones and text messaging to videos and online platforms (Kreuze et al., 2017). Technology may be used to supplement or adjunct an in-person intervention or may be the stand-alone component of the intervention (i.e., an automated intervention with no provider interaction) (Hermes et al., 2019). Hermes et al. (2019) call this range in technological use the “continuum of behavioral intervention technologies (BITs).” In contrast to other mental and behavioral health fields (Nguyen et al., 2013; Glover et al., 2019), suicidology, overall, does not currently use the BIT terminology to categorize how the role of technology differs across the delivery of technology-enhanced youth interventions.

While randomized controlled trials (RCTs) are often the gold standard for testing the efficacy of interventions (Hariton and Locascio, 2018), there is minimal knowledge on if and/or how trials incorporate implementation outcomes regarding youth suicide interventions. Implementation science is the study of methods to promote uptake of evidence-based practices and policies into the broader health care system (National Cancer Institute, 2020), and implementation is a critical element to understanding how and why technology-enhanced interventions work (or need adjustments) in the real world (Wozney et al., 2018). The implementation of health and mental health services is often measured by 8 defined outcomes: acceptability, adoption, appropriateness, feasibility, fidelity, implementation, penetration, and sustainability (Proctor et al., 2011). Without detailed measurement and assessment of these outcomes, pitfalls are not identified, and the implementation of technology-enhanced interventions in larger mental health care systems may fail (Graham et al., 2019). For example, concerns regarding user privacy and confidentiality, and the commercialization of mobile health tools may impact youth usage and the sustainability of interventions (Struik et al., 2017; Lustgarten et al., 2020). To address the limitations of RCTs, scholars now recommend the use of effectiveness-implementation hybrid designs, which integrate the testing or observation of the intervention's clinical impact and implementation processes (Curran et al., 2012).

### Objectives

In this review, we incorporate work from implementation science (Proctor et al., 2010, 2011) and BIT implementation (Wozney et al., 2018; Hermes et al., 2019) to illustrate how the study of technology-enhanced interventions for youth suicide can be advanced by specifying the role of technology and measuring diverse outcomes that take into account the unique features of this implementation context. Examples are drawn from 12 RCTs that were identified as part of a larger systematic review focused on the efficacy and effectiveness outcomes of technology-enhanced suicide interventions for youth (Szlyk and Tan, 2020). The 12 international studies represent interventions conducted over the last 19 years that span common place-based settings for youth (e.g., schools, hospital, clinics) and include online platforms as the treatment setting. The variety of selected studies demonstrate opportunities for innovation in measuring implementation outcomes and exemplify the heterogeneous use of technology in interventions.

In the following sections, we discuss how (1) how technology-enhanced interventions for youth suicide can be classified using the BIT continuum, and (2) how implementation outcomes can be measured in future effectiveness trials of these interventions. Our overall intent is to illuminate how technology-enhanced interventions for youth suicide can benefit from the explicit measurement of implementation outcomes.

## Redefining Implementation Outcomes for Technology- Enhanced Interventions for Youth Suicide

Proctor et al. (2011) taxonomy provided an invaluable foundation for measuring implementation outcomes in the behavioral health sciences. Currently, researchers suggest that the traditional implementation outcomes be redefined to account for the growing use of technology in behavioral and mental health interventions (Wozney et al., 2018; Hermes et al., 2019). For example, the meaning of traditional implementation outcomes may be different within the context of technology. Perceived usability and usefulness are considered other terms for feasibility and appropriateness; yet, in this context, they may be a better fit for measuring the acceptability of a technology-enhanced intervention (Brooke, 1996; Hermes et al., 2019). Also, since provider and organizational interaction can vary with technology-enhanced interventions, the level of analysis of implementation outcomes may be different as compared to face-to-face interventions. For instance, a fully-automated intervention will not measure implementation outcomes at the provider level, since interaction is between the youth consumer and tool or platform.

Now is an ideal moment for suicidologists to learn from colleagues who specialize in implementation science and BITs: researchers are rapidly developing new technology-enhanced interventions, there is a need for youth suicide interventions that are engaging and accessible, and researchers must also identify how current interventions can be improved and implemented into real-world practice settings. Keeping these three points in mind, we examined 12 RCT studies that included prominent youth suicide interventions that incorporated technology. The selected studies were the only RCTs identified through a large systematic review of the technology-enhanced interventions for youth suicide. The systematic review adhered to PRISMA guidelines (Moher et al., 2009) (please see flow diagram, search terms, and checklist in Supplementary Materials). Even though the large systematic review allowed for interesting insights about the technology-enhanced interventions, we felt that only an examination of the cumulative 26 studies (of varying study design) was a missed opportunity to understand the nuances of study subsamples (e.g., RCTs). We considered the identified RCTs to be an appropriate sample to examine how established and prominent youth suicide preventive interventions could be categorized along the BIT continuum and could measure implementation outcomes in future hybrid trials.

The interventions represented were: Signs of Suicide (SOS), a universal prevention program for high school (Aseltine and Demartino, 2004) and middle school students (Schilling et al., 2014); Brief Intervention and Contact (BIC; Bertolote et al., 2010); MI-SafeCope (Czyz et al., 2019), a motivational interview-enhanced safety planning intervention; ProHelp (Han et al., 2018), a brief psychoeducational online program; ReFrame-IT (Hetrick et al., 2017), an internet-based CBT program; Electronic Bridge to Mental Health Services (eBridge; King et al., 2015), which provided personalized feedback and optional online counseling for university students; the Youth Nominated Support Team Version Two (YST-II; King et al., 2009), a psycho-education and follow-up intervention following psychiatric hospitalization; Dialectical Behavior Therapy for Adolescents (DBT-A; Mehlum et al., 2014); the Family Intervention for Suicide Prevention (FISP; Rosenbaum et al., 2011), an adaptation of an emergency room intervention; Sources of Strength (Wyman et al., 2010), a school-based suicide prevention program; Coping Long-Term with Active Suicide Program for Adolescents (CLASP-A; Yen et al., 2019), an adapted program for the post-discharge transition period. Table 1 provides additional study characteristics.

**Table 1:** Study characteristics.

	**Country**	**Setting**	**Sample size**	**Mean age (years)**	**Majority gender of sample**	**Majority ethnicity of sample**	**Intervention name**
Aseltine and Demartino (2004)	USA	High School	2,100	Not Available	Female	Hispanic-non White	SOS
Bertolote et al. (2010)	Brazil; India; Sri Lanka; Iran, China	Emergency Department	1,867	23	Female	Indian	BIC
Czyz et al. (2019)	USA	Hospital	36	15.42	Female	White/ Caucasian	MI-SafeCope
Han et al. (2018)	Australia; China	University; online	257	19.32	Female	Chinese	ProHelp
Hetrick et al. (2017)	Australia	High School; online	50	14.7	Female	Not available	Reframe-IT
King et al. (2015)	USA	University; online	76	22.9	Female	White/ Caucasian	*e*Bridge
King et al. (2009)	USA	Hospital	448	15.59	Female	White/ Caucasian	YST-II
Mehlum et al. (2014)	Norway	Psychiatric Outpatient	77	15.6	Female	Norwegian	DBT-A
Rosenbaum et al. (2011)	USA	Emergency department	181	14.7	Female	Hispanic-non White	FISP
Schilling et al. (2014)	USA	Middle school	386	Not available	Female	White/ Caucasian	SOS-Middle School
Wyman et al. (2010)	USA	High school	2,675	Not available	Female	White/ Caucasian	Sources of Strength
Yen et al. (2019)	USA	Psychiatry inpatient unit	50	15.74	Female	White/ Caucasian	CLASP-A

### BIT Continuum and Potential Data Streams

Hermes et al. (2019) posit that the measurement of implementation outcomes needs to take into account the data streams of a BIT (how data is recorded and collected) and the continuum of provider and technology-based support (adjunct, guided, or fully-automated intervention). This contrasts the usual practice in suicidology, of categorizing and grouping interventions by tier of prevention: universal, selective, and indicated. For example, from this review: Sources of Strength (Wyman et al., 2010) is a universal preventive intervention that is offered to all students; eBridge (King et al., 2015) is a selective preventive intervention used to identify university students at elevated risk of suicide; DBT-A (Mehlum et al., 2014) is an indicated preventive intervention that treats youth experiencing severe suicidality. Prior to identifying implementation outcomes, we categorized the 12 studies as adjunctive, guided, or fully-automated (Hermes et al., 2019) and the use of technology (the data stream) was documented (see Table 2).

**Table 2:** The Continuum of Behavioral Intervention Technology (BIT) and use of technology.

	**Adjunctive** **BIT**	**Technology** **use**	**Guided BIT**	**Technology** **use**	**Fully automated BIT**	**Technology use**
	BIT supplements or enhances provider-delivered intervention		Key aspects of intervention delivered by BIT with provider support		BIT delivered intervention directly to consumer; minimal provider support	
Aseltine and Demartino (2004)	X	Psychoeduc-ational elements taught using video				
Bertolote et al. (2010)	X	Follow-up contacts included phone calls				
Czyz et al. (2019)	X	Check-in using phone call and text messages during follow up period				
Han et al. (2018)					X	Self-directed, online psychoeducat-ional program
Hetrick et al. (2017)			X	Online CBT modules delivered and supported by school well-being staff		
King et al. (2015)			X	Online screening program with counselor interaction		
King et al. (2009)	X	Phone consultation for the adult support persons post-discharge				
Mehlum et al. (2014)	X	Telephone coaching to support in-person DBT sessions				
Rosenbaum et al. (2011)	X	Telephone contacts for supporting outpatient treatment attendance post ED-discharge				
Schilling et al. (2014)	X	Psycho-education taught via DVD				
Wyman et al. (2010)	X	Use of videos, social networking sites, and text-messages to engage youth				
Yen et al. (2019)	X	Weekly telephone booster calls and daily text messages to enhance treatment engagement				

*Definition of BIT categories from Hermes et al. (2019)*.

Nine studies described interventions that could be categorized as adjunctive technology-enhanced interventions (Aseltine and Demartino, 2004; King et al., 2009; Bertolote et al., 2010; Wyman et al., 2010; Rosenbaum et al., 2011; Mehlum et al., 2014; Schilling et al., 2014; Czyz et al., 2019; Yen et al., 2019). This means that these studies featured a technological component to supplement an intervention delivered by a provider (Hermes et al., 2019). For example, the SOS program for high school students included a video to enhance a psychoeducational presentation about youth suicide risk (Aseltine and Demartino, 2004). Two studies described interventions that could be categorized as guided technology-enhanced interventions (King et al., 2015; Hetrick et al., 2017). Therefore, important aspects of the intervention were delivered by a technological component with some provider support. For instance, college students engaged the eBridge platform remotely, and mental health providers responded accordingly to students' completed suicide risk assessments and digital messages (King et al., 2015). Finally, one study described an intervention that could be categorized as a fully-automated technology-enhanced intervention (Han et al., 2018); college students accessed ProHelp, a brief psychoeducational online program, with minimal to no provider support. Technology used ranged from phones (either for calls or text messaging), videos, to online platforms, which suggests a potential variety of data streams.

### Measurement of Implementation Outcomes

We examined how the eight implementation outcomes from Proctor's taxonomy, with adjustments based on the work of Hermes et al. (2019) and Wozney et al. (2018) and colleagues, could be identified and measured among our sample (see Table 3). Feasibility was conceptualized as “trialability” and informed by recruitment, retention, and youth participation rates (Proctor et al., 2011). Feasibility was measured by the number or proportion of youth participants recruited, enrolled, and retained in the study or lost to dropout. Eight studies measured adoption of the intervention (Aseltine and Demartino, 2004; King et al., 2009, 2015; Wyman et al., 2010; Mehlum et al., 2014; Hetrick et al., 2017; Czyz et al., 2019; Yen et al., 2019), and, overall, studies recorded session attendance and participant engagement with online intervention tools and modules. Five studies measured intervention fidelity by using adherence rating scales for session evaluation (King et al., 2009; Mehlum et al., 2014; Czyz et al., 2019; Yen et al., 2019), interrater reliability of psychometric outcomes and session checklists (King et al., 2009) and verification of completion of intervention components by additional sources (Wyman et al., 2010).

**Table 3:** Implementation outcomes reported and how they were measured.

	**Acceptability**	**Adoption**	**Appropriateness**	**Cost**	**Feasibility**	**Fidelity**	**Penetration**	**Sustainability**
	Satisfaction with aspects of the intervention	Uptake; intention to use	Perceived fit, relevance or compatibility of the intervention	Cost of develop-ment and implement-tation effort	Extent to which the intervention can be carried out	Adherence to original intervention as intended	Integration of intervention within system	Extent to which the intervention is maintained within the system
Aseltine and Demartino (2004)	^*^Earlier publication reported school staff's perception of the satisfaction of intervention components and materials to students	^*^Reported teachers' summary of student participation at video screenings	^*^School staff rated perceived helpfulness and practicability of intervention components and materials		Sample number engaged in study and lost to dropout			
Bertolote et al. (2010)					Number of participants lost to follow-up			
Czyz et al. (2019)	Youth and parents rated general satisfaction with the intervention and if they would recommend it to others	Youth's daily engagement in coping skills and safety planning skills acquired during intervention			Percentage of participants enrolled, completion of components and participation in follow-up	Adherence measure, by intervention counselor during or after sessions		
Han et al. (2018)	Internet Evaluation and Utility Question-naire (IEUQ)^a^ used to assess youth's usability, likeability, and usefulness; also questions about ease of use and clarity of information provided.		Youth's professional help-seeking beliefs items based on General Help-Seeking Question-naire^b^ Youth's professional help-seeking attitudes were measured by the Attitudes Toward Seeking ProfessionalPsychological Help Scale^c^		Counted clicks on Facebook advertisement and number of students invited from the SONA platform. Number of participants recruited, eligible, and engaged in study. Completion rates for post-test and 1-month follow-up surveys.			
Hetrick et al. (2017)		Metrics of how many modules and how much of each module was completed by youth, how many activities were completed, and how often the message board was used.			Sample number engaged in study, lost to dropout, and completion of follow-up assessments			
King et al. (2015)		Number of participants who sent messages to the counselor (i.e., never, once, etc.); number of participants who viewed feedback from counselor	Need for help, assessed if in the previous 2 months the youth thought they needed help for emotional, mental health or problems related to substance abuse. Readiness to access help was assessed, with responses: “Sometimes I think about doing this”; “I have taken steps toward doing this”; and “I already did this.”		Number of participants enrolled and number of participants retained at follow-up assessment.			
King et al. (2009)		Number of sessions attended by parent/adult support person; number of calls and face to face interactions; percentage of participants using treatment method at stages of study			Retention of sample at each time point in the study.	Intervention sessions were audiotaped and specialists completed checklists after sessions. Interrater reliability was established on psycho-metric outcomes and session categories.		
Mehlum et al. (2014)		Mean scores of all participant completion of sessions by modality (i.e., individual or family therapy)			Number of participants enrolled and number lost to dropout; ^*^more than 3 dropped individual therapy sessions is considered dropout in DBT-A.	Adherence was assessed by an independent rater using the DBT Global Rating Scale^d^. For each patient-therapist dyad individual therapy, 5 sessions were videotaped. One randomly selected videotaped skills training session per group was rated per month.		
Rosenbaum et al. (2011)					Number of participants enrolled and lost to dropout			
Schilling et al. (2014)			Eight questions were used to assess participants' help-seeking behavior.		Number of schools approached for study and number enrolled; number of participants who returned consent forms; number of participants enrolled.			
Wyman et al. (2010)		Peer leaders completion of the messaging steps of the intervention			Number of participants enrolled and number who completed pre and post-tests.	Staff members were interviewed after the messaging phase to verify peer leaders' compliance.		
Yen et al. (2019)	Participants and parents provided intervention approval ratings on a Likert scale; open-ended comments recorded.	Number of sessions completed by participant and parents			Number of participants enrolled, retainment at study stages, and number lost to dropout.	A blind independent evaluator (pre-doctoral fellow) rated session tapes for adherence and competency.		

* *See Aseltine (2003).*

a *IEUQ Information: 15 items with 2 open-ended questions (Ritterband et al., 2008; Thorndike et al., 2008).*

b *General Help-Seeking Questionnaire (GHSQ; Wilson et al., 2005).*

c *Attitudes Toward Seeking Professional Psychological Help Scale (ATSPPHS-SF; Fischer and Farina, 1995).*

d *DBT Global Rating Scale Information: (Linehan, 2003), a 64-item instrument scored from 0 to 5, with higher scores reflecting higher adherence*.

Four studies measured acceptability of the intervention and its components by using participant ratings or responses to open-ended questions about intervention satisfaction (Aseltine, 2003; Han et al., 2018; Czyz et al., 2019; Yen et al., 2019). One study also measured acceptability by using the Internet Evaluation and Utility Questionnaire (IEUQ; Ritterband et al., 2008; Thorndike et al., 2008), which assesses usability, likeability, and usefulness of an online intervention (Han et al., 2018). Four studies measured appropriateness of the intervention by assessments of participants' help-seeking behaviors (Schilling et al., 2014) and beliefs and attitudes about help-seeking (Han et al., 2018), perceptions of intervention helpfulness and practicability (Aseltine, 2003), and ratings of perceived need for help and readiness to access help (King et al., 2015). We included measures of help-seeking as a proxy for appropriateness since this key behavior influences the intervention's fit and relevance for youth participants struggling with suicidality.

Examples of implementation cost, penetration, and sustainability were not identified among our sample. It is logical that these three outcomes were not identified among the RCTs, as they are outcomes that are observed or that occur in later stages of implementation (Proctor et al., 2011). Suggestions for future research are outlined in the discussion section.

## Discussion

This review sought to discuss and demonstrate how technology-enhanced interventions for youth suicide can adopt the terminology of the BIT continuum and begin to measure implementation outcomes in future hybrid trials. Based on the first exercise, technology-enhanced interventions for youth greatly varied in terms of provider and technology support. Therefore, the conceptualization of implementation outcomes and how they can be measured or observed should be specific to the category of the BIT continuum. For instance, the SOS program (Aseltine and Demartino, 2004; Schilling et al., 2014), an adjunctive BIT, would measure implementation outcomes differently than ProHelp (Han et al., 2018), a fully-automated BIT. The SOS program could measure implementation outcomes by different levels of analysis—provider (teacher or mental health professional), consumer (student), and administrator (principal), while ProHelp may only report consumer-based outcomes (the youth who are accessing the intervention).

Additionally, we discovered that data stream sources range across the BIT continuum and within BIT categories. Using the same example, the SOS program's psychoeducation video cannot be used as a source of implementation data collection, while ProHelp's online platform automatically records youth intervention engagement and use (i.e., youth clicks on an online advertisement, participant entry into the platform). Yet, MI-SafeCope (Czyz et al., 2019), another adjunctive BIT, had a different data stream than the SOS program by using phone calls and text messages for participant follow-up. Therefore, technology-enhanced suicide interventions for youth are incredibly heterogeneous, and suicidologists should consider an intervention's stage in the BIT continuum and data stream when measuring both effectiveness and implementation outcomes. These observations may inform how specifically technology-enhanced interventions should be matched when comparing outcomes, since comparison by common characteristics (e.g., sample population, study design) overlooks that the interventions' mechanisms are extremely varied.

It is important to note that the majority of our sample were adjunctive technology-enhanced interventions. This is likely the result of the elongated time frame it takes to pilot, adapt, and then perform an RCT using a then considered “cutting edge technology” (e.g., text-messaging, DVDs). We believe more RCTs will be conducted for guided and automated interventions, as technology progresses, and researchers and funders become more adept at expediting the experimental process for technology-enhanced interventions.

The second exercise demonstrated how implementation outcomes can be potentially measured in RCTs of technology-enhanced interventions for youth suicide. Of the constructs present, outcomes were measured via observations or counts, questionnaires and/or rating. Future studies may benefit from including case audits, analyses of administrative data, qualitative methods (focus groups and semi-structured interviews), and the leveraging of data that platforms automatically collect (Proctor et al., 2011; Hermes et al., 2019) and balance outcomes reported by youth consumers and parents/guardians, non-clinical actors (such as peer leaders and school staff), clinical providers, and organizational administrators (Wozney et al., 2018). The practice of collecting implementation outcomes from various sources may also help to ensure that new interventions adequately respect youth privacy and confidentiality, and that user data is managed accordingly.

Researchers should consider measuring and reporting implementation cost, penetration, and sustainability, as these outcomes are also dependent on a study's stage in the BIT continuum, source of data stream, setting, and stage in the implementation process. Implementation cost is an outcome that is relevant to all stages of implementation (Proctor et al., 2011), and increased reporting of cost can inform colleagues and funding sources of the financial realities of developing, disseminating, and sustaining a technology-enhanced intervention for youth suicide. For instance, interventions that have more provider-driven components (such as DBT-A; Mehlum et al., 2014) would require substantial funding for ongoing clinician trainings, while interventions with more automated components (such as Reframe-IT; Hetrick et al., 2017) would require funding for launching, monitoring, and maintaining an online platform. Researchers must also consider the potential financial cost for the youth user (e.g., payment for internet access or cellphone coverage, payment to access mobile app).

Guided or fully-automated interventions broaden the possibilities for measuring penetration, since interventions can be disseminated or accessed by many people at a faster rate than interventions that are mainly face to face. This also suggests that the level of analysis may now include a virtual setting that is part of a larger education or medical institution or under the auspice of a private technological software company. For example, the school-based SOS program (Aseltine and Demartino, 2004) (an adjunctive BIT) would measure penetration by including school personnel and student peers who are trained and who implement the intervention (level of analysis at the school or school district); eBridge (King et al., 2015) (a guided BIT) may measure penetration based on the online platform's capacity to screen a certain number of students by the number of students who access the platform (level of analysis at the university counseling center's platform). Lastly, measurement of sustainability may now include the technological evolution of a youth suicide intervention. For instance, a fully-automated intervention would track updates (e.g., version 2.0 of a mobile application), and report adjustments to account for new technology and consumer preferences (e.g., switching from having the intervention developed for a mobile application specific to Android phones to one featured on IPhones).

### Conclusion

Overall, this review emphasizes the diversity within the sub-field of technology-enhanced interventions for youth suicide. Therefore, it is important that suicidologists be specific of how their intervention uses technology, varies in provider and technology-based support, and measures implementation outcomes. As youth suicide and suicidality continues to increase in the U.S., especially among youth with minority identities, the measurement of implementation outcomes may help to understand why an intervention fails or underperforms among a certain youth population, and how successful interventions can be disseminated more broadly. This review also illustrates that implementation outcomes can be measured as early as the RCT phase and raises considerations for how outcomes could be integrated in more implementation-focused studies.

The ever increasing integration of technology in interventions provides opportunities to innovate youth engagement and access. Yet, it also provides opportunities for further stigmatization of underserved populations and misallocated efforts if interventions do not take into account the needs of youth and providers, and the realities of implementing interventions beyond controlled settings. As demonstrated by colleagues specializing in implementation science and BIT, the development and testing of technology-enhanced interventions for youth suicide allow for recharacterization of implementation outcome measurement and, thus, heighten chances of achieving public health impact (Graham et al., 2019). We hope that this review honors the work of youth suicide researchers who have integrated technology into interventions and inspires future suicidologists to understand the nuances of technology-enhanced interventions, and how both provider and technologically-based components translate to implementation in real-world settings.

## Author Contributions

HS and JT identified the subsample of articles and extracted examples based on implementation and Behavioral Intervention Technology (BIT) frameworks. RL-H provided expertise in implementation science to evaluate examples identified from selected studies. HS wrote body of manuscript. JT and RL-H edited final versions of manuscript. All authors contributed to the article and approved the submitted version.

## Conflict of Interest

The authors declare that the research was conducted in the absence of any commercial or financial relationships that could be construed as a potential conflict of interest.
