# Supplementary material for: Innovating Technology-Enhanced Interventions for Youth Suicide: Insights for Measuring Implementation Outcomes
Source: Front Psychol. 2021 Jun 3;12:657303. doi: 10.3389/fpsyg.2021.657303 (PMC8210584; doi:10.3389/fpsyg.2021.657303)
Supplement: Supplementary file 1 [file Table_1.docx]

Figure 1. *PRISMA diagram for search strategy.*

Records identified through database searching
(n =943)

Additional records identified through other sources
(n =17)

Records after duplicates removed
(n =189)

Records screened
(n =771)

Excluded
(n =614)

Excluded (n =130)

Not an evaluation of intervention = 38

Only provided qualitative findings = 1

Earlier version of same study= 7

Not original research = 16

Did not meet age requirement = 45

Not primarily focused on suicidality =13

Primarily focused on NSSI= 3

Study protocol only = 3

Gatekeeper outcomes only =2

Legal case = 1

Psychometrics of assessment only = 1

Full-text articles assessed for eligibility
(n =157)

Full-text article excluded, during abstraction (earlier trial of an identified study); non-RCTs
(n =15)

Studies included in qualitative synthesis/abstraction
(n = 27)

Studies included in review
(n = 12)
