# Supplementary material for: Innovating Technology-Enhanced Interventions for Youth Suicide: Insights for Measuring Implementation Outcomes
Source: Front Psychol. 2021 Jun 3;12:657303. doi: 10.3389/fpsyg.2021.657303 (PMC8210584; doi:10.3389/fpsyg.2021.657303)
Supplement: Supplementary file 2 [file Table_2.docx]

LITERATURE SEARCH TRACKING FORM

Ovid Medline

Date Searched: 4/5/19

Applied Database Supplied Limits: English

(exp Young Adult/ OR exp Adolescent/) OR (Adolescent* OR high school OR college OR university OR youth* OR Young adult* OR emerging adult* OR teen*).mp.) AND

(exp Hotlines/ OR exp Telemedicine/ OR exp Social Media/) OR (Teleconsultation OR telehealth OR telemedicine OR mhealth OR mobile health OR video conference* OR remote consultation* OR web 2 OR social media OR social medium OR twitter OR facebook OR Instagram OR tumblr OR reddit OR ebridge OR online OR electronic bridge).mp.

AND

(exp Suicide/ OR Suicid*.mp.)

NOT (systematic review OR newspaper OR meta analysis OR editorial OR book review).pt.

PsycINFO

Date Searched: 4/5/19

Applied Database Supplied Limits: excluded non-English and reports that were CEUs or magazine articles or electronic collections (e-resources)

Full Search Strategy:

DE "Suicide" OR DE "Attempted Suicide" OR DE "Suicidality" OR suicid*

AND

DE "Telemedicine" OR DE "Teleconferencing" OR DE "Online Therapy" OR DE "Teleconsultation" OR DE "Telepsychiatry" OR DE "Telepsychology" OR DE "Telerehabilitation" OR DE "Social Media" OR DE "Online Social Networks" OR Teleconsultation OR telehealth OR telemedicine OR mhealth OR “mobile health” OR “video conference*” OR “remote consultati*” OR “web 2.0” OR “social media” OR “social medium” OR “twitter” OR “facebook” OR “Instagram” OR “tumblr” OR “reddit” OR DE "Hot Line Services"

AND

DE "Colleges" OR DE "High Schools" OR DE "Emerging Adulthood" OR "adolescent" OR DE "Middle School Students" OR “young*”

CINAHL

Date Searched: 4/5/19

Applied Database Supplied Limits: excluded non-English and reports that were CEUs or magazine articles

Full Search Strategy:

((MH "Telemedicine+") OR (MH "Telepsychiatry") OR (MH "Text Messaging") OR (MH "Instant Messaging") OR (MH "Internet") OR (MH "Videoconferencing") OR (MH "Social Media") OR (MH "Online Services") OR (MH "Online Social Networking") OR ("online therapy") OR (MH "Telephone Information Services") OR ("hotline"))

AND

(MH "Suicide+" OR suicide* OR suicidal*)

("adolescent") OR (MH "Young Adult") OR (MH "Students, High School") OR (MH "Students, Middle School") OR (MH "Students, College") OR "youth" OR "young person" OR "teen*"

ClinicalTrials.gov

Date Searched: 4/8/19

Terms entered int Clinical Trials search engine. We completed 8 separate searches on this site, using the following terms:

Condition or Disease: Suicidal and Self-injurious Behavior AND Technology (other term)

Condition or Disease: Suicidal and Self-injurious Behavior AND mobile (other term)

Condition or Disease: Suicidal and Self-injurious Behavior AND online (other term)

Condition or Disease: Suicidal and Self-injurious Behavior AND hotline (other term)

Condition or Disease: Suicidal and Self-injurious Behavior AND text (other term)

Condition or Disease: Suicidal and Self-injurious Behavior AND video (other term)

Condition or Disease: Suicidal and Self-injurious Behavior AND tele (other term)

Condition or Disease: Suicidal and Self-injurious Behavior AND social media (other term)
